# Long-term (2001–2013) observations of water-soluble dicarboxylic acids and related compounds over the western North Pacific: trends, seasonality and source apportionment

**DOI:** 10.1038/s41598-017-08745-w

**Published:** 2017-08-17

**Authors:** Suresh K. R. Boreddy, Kimitaka Kawamura, Eri Tachibana

**Affiliations:** 1 Institute of Low Temperature Science, Hokkaido University, N19, W8, Kita-Ku, Sapporo, 060-0819 Japan; 2 Chubu Institute for Advanced Studies, Chubu University, 1200 Matsumoto- cho, Kasugai, 487–8501 Japan

## Abstract

To better understand the impact of East Asian pollutants on the molecular composition of marine organic aerosols, we conducted long-term (2001–2013) observations of water-soluble dicarboxylic acids and related compounds in total suspended particulate samples collected at Chichijima Island in the western North Pacific (WNP). Seasonal variations of all the diacids and related compounds showed maxima in winter and spring and minima in summer, except for azelaic acid (C_9_), which maximized in summer to autumn. The overall annual concentrations of the total diacids, ω-oxoacids and α-dicarbonyls showed an increase during 2001–2013. We found a significant (p < 0.05) decadal increase in the inter-annual trends of pyruvic and glyoxylic (p > 0.05) acids, and methylglyoxal (MeGly). In contrast, phthalic acid (p < 0.05) and glyoxal (Gly) showed a decrease in their trends. We also found a significant decrease in the trend of the Gly/MeGly mass ratios. These results demonstrate that the enhanced concentrations of diacids over the WNP are majorly attributed to the aqueous-phase photooxidation of biogenic volatile organic compounds from East Asia followed by long-range atmospheric transport. Further, positive matrix factorization analysis showed a biogenic photochemical contribution (42%) was the dominant source of oxalic acid in the WNP.

## Introduction

Low molecular weight dicarboxylic acids (diacids) are ubiquitous and important constituents of atmospheric organic aerosols^1^. Due to the low vapor pressures (approximately less than 10^−7^ Pa)^2, 3^, these compounds are almost exclusively partitioned into a particulate phase and contribute significantly to the water-soluble fraction^4, 5^. Thus, particles enriched with diacids can act as cloud condensation nuclei^6–8^ and have an impact on the earth’s radiative forcing^9^. Diacids also play an important role in many biological processes in the ocean^10^. Despite the great importance, they continuously represent a challenge to atmospheric scientists due to their complexity, although the first unambiguous observations of diacids in the atmosphere were made three decades ago^11^.

Diacids are the late photooxidation products of hydrocarbons and other organics^12^. Therefore, photochemical chain reactions are the most important sources of diacids^13, 14^. Aqueous-phase oxidation and cloud processing of aerosols are potentially important sources of organic aerosols and could explain the high abundances of oxalic acid in the atmosphere^15–25^. High levels of diacids were observed in motor exhausts and ambient aerosols influenced by forest fires^26, 27^, indicating important primary sources of diacids. However, there is still a knowledge gap on the relative contribution of primary and secondary sources of diacids in the atmosphere. On the other hand, iron-carboxylate complexes and their photochemical degradation in the water mediated atmosphere^28–32^ may be an important sink of diacids in addition to wet and dry deposition.

A large fraction of secondary organic aerosol (SOA) is derived from the oxidation of isoprene, monoterpenes and sesquiterpenes of biogenic origin. Global aerosol modelling studies suggest an increase in isoprene emissions of 22 to 55% by 2100 in response to the temperature increase following a global warming^33–35^. Recent modelling studies^36^ simulate an increased trend of isoprene emissions with 0.16% per year over Asia and this trend is more pronounced over China with 0.52% per year during 1979 to 2012. All these models, however, have significant uncertainties in the projection of SOA formation due to a series of fundamental assumptions and lack of long-term observational studies. The models assume that particles are liquids and are exposed to changes in the surrounding atmosphere by rapid condensation or evaporation and by in-particle mixing during long-range atmospheric transport^37^.

East Asia is one of the most populated regions on the globe with a population density of 133 inhabitants per square kilometer, which is about three times higher than the world population density (45 per km^2^). Increasing levels of air pollutants in East Asia and their outflows to the Pacific have received a significant attention due to the potential impacts on regional and global climate and atmospheric circulations^38–43^. However, the effects of East Asian pollutants and their outflows over the western North Pacific (WNP) are still unclear, especially with regard to the sources and formation of SOA during long-range transport^44–46^.

In this study, we carried out long-term (2001–2013) measurements of diacids and related compounds in total suspended particulate (TSP) aerosols collected at Chichijima Island in the WNP. Chichijima is a remote island 2000 km away from East Asia and exists in the outflow region of Asian dusts and pollutants from East Asia, especially China. Here, we discuss the inter-annual trends and seasonality of diacids over the WNP. We also discuss the sources and formation pathways of the diacids using a positive matrix factorization (PMF) model.

## Results and Discussion

### Air mass origin and meteorological parameters

**Figure 1:**
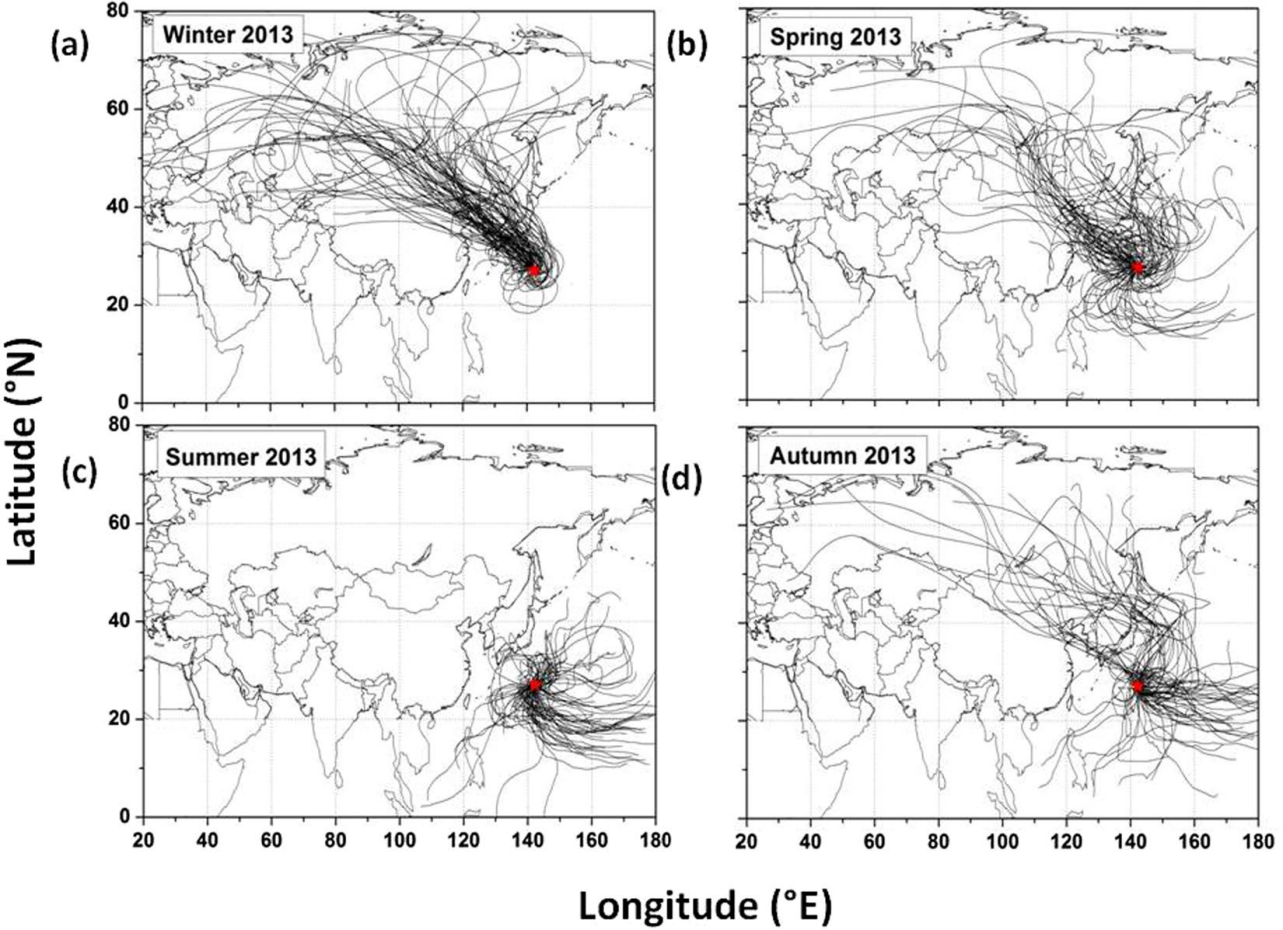
Daily 7-day HYSPLIT backward air mass trajectories for different seasons over the WNP for the year 2013. The star (*) indicates the sampling site, Chichijima Island. Trajectory data downloaded from the NOAA ARL website (http://www.arl.noaa.gov) and plotted using origin lab (v.8) software (http://www.originlab.com/).

Figure 1(a–d) presents daily 7-day isentropic air mass back trajectories at an altitude of 500 m above the ground level using the HYSPLIT model^47^ for different seasons during the year 2013 over the WNP as an example. Air mass transport from East Asia to the sampling site in the Pacific is stronger during winter (December to February) and spring (March to May) than during summer and autumn to deliver continental air masses via long-range atmospheric transport. The continental air mass transport is almost absent in summer (June to August). Air masses mostly come from the central Pacific carrying pristine air masses to the observation site in summer, whereas in autumn (September to November) the air mass pathway shifts from southeasterly to northwesterly towards winter. More detailed information of air mass transport over the WNP is described elsewhere^48^.

The meteorological parameters such as air temperature (°C), wind speed (ms^−1^), solar radiation (MJ m^−2^), precipitation (mm) and cloud cover are downloaded from the Japan Meteorological Agency (JMA) for Chichijima Island during 2001–2013 (Figure S1). We found a clear seasonal variation in air temperature, solar radiation and precipitation with summer maxima and winter minima. Wind speed and cloud cover were higher in spring and lower in summer. However, no significant decadal trends were observed in any meteorological parameters throughout the sampling period, therefore, we used meteorological parameters in this study to better understand the seasonal variations of organic compounds but not to discuss the trends.

### Molecular distributions

In order to better understand the sources, formation pathways, and physicochemical properties of organic aerosols, we studied the molecular distributions of the diacids and related compounds at Chichijima Island between 2001 and 2013. Molecular distributions are shown in Figure S2. Throughout the observation period, we found the predominance of oxalic acid (C_2_) followed by malonic (C_3_) and/or succinic (C_4_) acids. This molecular distribution is consistent with our previous study for 1990–1993 at the same observation site^44^ and other East Asian sites such as Okinawa Island^49, 50^, the Gosan site, Jeju Island in South Korea^51^, Mt. Tai in North China^52^, urban sites in China^53, 54^, as well as different sites in the world including Tanzania, East Africa^55^, remote western European continental sites^56^, west-east transect in the European atmosphere^57^, and Los Angeles^26^.

Glyoxylic acid (ωC_2_) was the dominant species among all ω-oxoacids and the fourth most abundant species detected, whereas methylglyoxal (MeGly) was more abundant than glyoxal (Gly) in the WNP aerosols. Throughout the observation period, we found the molecular distributions of the diacids and related compounds at Chichijima as C_2_ > C_3_ > C_4_ > ωC_2_ > MeGly > Ph > ωC_7_ > C_5_ > ωC_8_ > Gly > C_6_ > ωC_9_ > C_9_. However, seasonal molecular distributions provided a different picture depending on the source strengths and formation mechanisms.

### Trends

**Figure 2:**
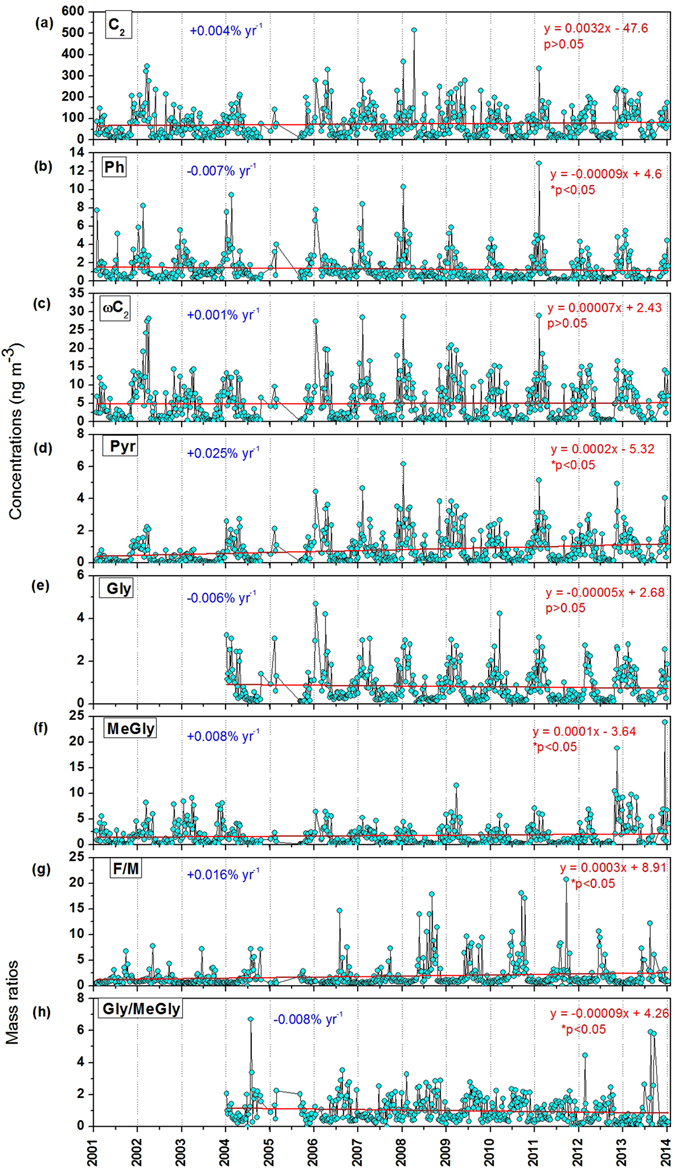
Trends in (**a**–**f**) temporal variations of water-soluble dicarboxylic acids (ng m^−3^) and related compounds (ng m^−3^) and (**g**,**h**) F/M and Gly/MeGly mass ratios in TSP aerosols collected at Chichijima Island during 2001 to 2013. Linear regression trends are given inset and applied over the whole observation period. The symbol, *, indicates the trends are significant at a 95% (p < 0.05) confidence level. The solid red line represents the trend line.

Table 1 summarizes the regression statistics such as range, mean ± SD, and the trend (slope) for water-soluble diacids, ω-oxocarboxylic acids, pyruvic acid, α-dicarbonyls, and their diagnostic ratios during the period of 2001 to 2013. Figure 2 presents the temporal trends of the major diacids and related compounds and diagnostic mass ratios during the whole study period.

**Table 1:** Regression statistics (range, mean ± SD, and slope) of water-soluble dicarboxylic acids and related compounds (n = 607) in remote marine TSP aerosols collected at Chichijima Island during the period of 2001 to 2013.

Organic compounds	Range (mean ± SD)	Slope (m, diacid year^−1^)	Uncertainty (σm)	Trend (% year^−1^)
Dicarboxylic acids (ng m^−3^)				
Normal chain saturated diacids				
Oxalic, C_2_	2.21–514 (73.9 ± 66.8)	+0.0032	0.0020	+0.004
Malonic, C_3_	0.28–55.6 (11.5 ± 9.4)	+0.00005	0.0002	+0.0004
Succinic, C_4_	0.05–52.4 (6.12 ± 6.41)	+0.0002*	0.0002	+ 0.003
Glutaric, C_5_	0–7.48 (1.11 ± 1.19)	−0.00003*	0.00003	−0.0027
Adipic, C_6_	0.01–5.08 (0.59 ± 0.60)	−0.00002	0.0000017	−0.0033
Pimelic, C_7_	0–1.34 (0.15 ± 0.16)	−0.000014*	0.0000047	−0.009
Suberic, C_8_	0–1.24 (0.13 ± 0.14)	−0.00003*	0.0000038	−0.023
Azelaic, C_9_	0.01–2.50 (0.53 ± 0.34)	−0.00003*	0.00001	−0.005
Decanedioic, C_10_	0–0.95 (0.06 ± 0.08)	−0.000003	0.0000023	−0.005
Undecanedioic, C_11_	0–8.03 (0.08 ± 0.34)	−0.000002	0.0000098	−0.002
Dodecanedioic, C_12_	0–0.34 (0.01 ± 0.04)	−0.000004*	0.000001	−0.04
Branched chain saturated diacids				
Methylmalonic, iC_4_	0–1.45 (0.31 ± 0.25)	−0.000009	0.0000074	−0.0029
Methylsuccinic, iC_5_	0–3.13 (0.52 ± 0.48)	+0.000012	0.000014	+0.0019
Methylglutaric, iC_6_	0–0.96 (0.08 ± 0.09)	−0.000006*	0.000002	−0.0075
Multi functional saturated diacids				
Hydroxysuccinic, hC_4_	0–15.2 (0.26 ± 1.12)	−0.000111*	0.000032	−0.038
Ketomalonic, kC_3_	0–5.40 (0.38 ± 0.54)	+0.00002	0.000015	+0.0052
Ketopimelic, kC_7_	0–3.67 (0.50 ± 0.57)	−0.000007	0.000017	−0.0014
Unsaturated aliphatic diacids				
Maleic, M	0–2 (0.43 ± 0.40)	−0.00005*	0.00001	−0.011
Fumaric, F	0–2.27 (0.41 ± 0.32)	−0.00002*	0.0000092	−0.005
Methylmaleic, mM	0–7.66 (0.22 ± 0.44)	−0.00007*	0.000012	−0.031
Aromatic diacids				
Phthalic, Ph	0.01–12.8 (1.31 ± 1.49)	−0.00009*	0.000043	−0.0068
Isophthalic, iPh	0–11.1 (0.14 ± 0.49)	+0.00001	0.000014	+0.0071
Terephthalic, tPh	0–7.18 (0.39 ± 0.52)	+0.00002	0.000015	+0.0051
* Total diacids*	2.93–555 (99.2 ± 86.4)	+0.003	0.0025	+0.003
ω-Oxocarboxylic acids (ng m^−3^)				
Glyoxylic, ωC_2_	0.09–29 (4.91 ± 5.13)	+0.00007	0.00015	+0.001
3-Oxopropanoic, ωC_3_	0–22.4 (0.26 ± 0.94)	+0.00006*	0.000027	+0.023
4-Oxobutanoic, ωC_4_	0–5.56 (0.39 ± 0.47)	+0.00004*	0.000013	+0.010
5-Oxopentanoic, ωC_5_	0–0.65 (0.09 ± 0.08)	+0.00001*	0.0000024	+0.01
7-Oxoheptanoic, ωC_7_	0.02–11.6 (1.13 ± 1.27)	+0.000006	0.000037	+0.0005
8-Oxooctanoic, ωC_8_	0–6.32 (0.72 ± 0.89)	+0.00002	0.000026	+0.0027
9-Oxononanoic, ωC_9_	0–5.72 (0.59 ± 0.68)	+0.00006*	0.00002	+0.010
* Total* ω-*oxoacids*	0.21–52.7 (8.10 ± 8.20)	+0.0003	0.00024	+0.0037
Ketoacid (ng m^−3^)				
Pyruvic, Pyr	0–6.16 (0.79 ± 0.90)	+0.0002*	0.000025	+0.025
α-Dicarbonyls (ng m^−3^)				
Glyoxal, Gly	0–4.69 (0.80 ± 0.78)	−0.00005	0.000036	−0.006
Methylglyoxal, MeGly	0–23.9 (1.73 ± 2.28)	+0.00014*	0.000067	+0.008
* Total α-dicarbonyls*	0.04–26.4 (2.34 ± 2.74)	+0.0003*	0.00008	+0.012
Ratios				
* F*/*M*	0–20.8 (1.83 ± 2.47)	+0.0003*	0.000072	+0.016
* C*_2_/*C*_3_	1.46–33.1 (6.55 ± 2.86)	+0.00034*	0.000083	+0.0045
* C*_2_/*C*_4_	0.80–154 (17.0 ± 12.3)	+0.001*	0.00036	+0.005
* C*_3_/*C*_4_	0.15–27.7 (2.76 ± 1.93)	+0.00002	0.000057	+0.0007
* Ph*/*C*_9_	0.01–77.0 (3.47 ± 5.10)	−0.000006	0.00015	−0.0002
* C*_2_/*∑*(*C*_2_ *-C*_12_)	0.38–0.94 (0.77 ± 0.06)	+0.000008*	0.0000017	+0.0010
* C*_2_/*ωC*_2_	5.48–131 (22.2 ± 14.7)	+ 0.0013*	0.0004	+0.0058
* C*_2_/*Gly*	20.3–4050 (135 ± 332)	+0.0022	0.01	+0.002
* C*_2_/*MeGly*	0–758 (90.8 ± 86.3)	+0.0067	0.0025	+0.007
* Gly*/*MeGly*	0–6.71 (0.98 ± 0.81)	−0.00008*	0.000037	−0.008

The symbol, *, indicates the trends are significant at a 95% (p < 0.05) confidence level.

C_2_, C_3_, and C_4_ acids are the end or near-end products of photochemical reaction chains of hydrocarbons and biogenic unsaturated fatty acids, accounting for approximately 74%, 11%, and 6% of the total diacids, respectively, during the 13-year study period at Chichijima. From Table 1, it is obvious that C_4_ shows an increasing trend (+0.003% yr^−1^), whereas those of C_5_–C_12_ diacids (particularly, C_5_, C_7_, C_8_, C_9_, and C_12_) show significant (p < 0.05) decreasing trends during the study period. These results suggest a photochemical conversion of higher to lower molecular weight diacids during the long-range atmospheric transport over the WNP. This point is further supported by significant increasing trends of diagnostic mass ratios of diacids, as discussed below.

It has been suggested that maleic acid (*cis* configuration) (M), a photo-oxidation product of aromatic hydrocarbons such as benzene and toluene^58^, may be isomerized to fumaric acid (*trans* configuration) (F) under a high solar radiation in the atmosphere^1^. Therefore, the F/M ratio may be a good indicator of photochemical processing. In our study, we found a significant (p < 0.05) increasing trend (+0.016% yr^−1^) of F/M ratios during 13 years of the study period (Fig. 2g), indicating an intensive photochemical aging or increased oxidant levels over the WNP. Further, it has been suggested that C_2_ and C_3_ are likely produced in the marine atmosphere by the photooxidation of C_4_ through intermediates such as hydroxylsuccinic or malic acid (hC_4_) and ketomalonic acid (kC_3_)^24, 59, 60^. In this study, we found that C_3_/C_4_ ratios showed an increasing trend, although it is not significant (p > 0.05). However, C_2_/C_3_ and C_2_/C_4_ ratios show increases (+0.004% yr^−1^ and +0.005% yr^−1^) during the 13 year study period. This observation suggests that C_2_ could be largely produced by the photochemical degradation of C_3_ and C_4_ diacids. A significant (p < 0.05) increase (+0.002% yr^−1^) of C_2_/Σ (C_2_–C_12_) further supports the photochemical aging of diacids over the remote marine aerosols (Table 1). All these results suggest that the production of diacids over the WNP may be closely linked with an increased photochemical oxidation of biogenic and anthropogenic precursors that are delivered from the Asian continent by long-range atmospheric transport.

Previous studies suggested that oxidation of aromatic hydrocarbons such as naphthalene and o-xylene, which originate from an incomplete combustion of fossil fuel^26^, is one of the major sources of phthalic acid (Ph) in the atmosphere^61, 62^. High abundances of aromatic hydrocarbons are reported over China during winter^63, 64^. Similarly, adipic acid (C_6_) is probably produced through the oxidation of cyclohexene by ozone in the atmosphere and has been proposed as an anthropogenic tracer^46^. It is also well documented that glyoxal (Gly) is largely produced in the atmosphere by the oxidation of many anthropogenic aromatic hydrocarbons^65–67^, although it has small contribution from biogenic and marine origin^20, 68, 69^. In this context, we found a decreasing trend in the concentrations of Ph (p < 0.05; −0.007% yr^−1^), C_6_ (p > 0.05; −0.003% yr^−1^) and Gly (p > 0.05; −0.006% yr^−1^) (Fig. 2b,e, and Table 1). These results suggest that combustion (fossil fuel) derived aerosols have declined (or constant) over the WNP during 2001–2013. This point is further supported by the study of Boreddy, *et al*.^70^, who reported that declined concentrations of elemental carbon (EC) over WNP occurred during 2001–2012 over the WNP. However, it should be noted that although Gly concentrations are decreased, its processing to C_2_^71^ is not decreased over the WNP, as evidenced by the increasing trend (+0.002% yr^−1^; p > 0.05) of C_2_/Gly ratios (Table 1).

On the other hand, a modeling study by Stavrakou, *et al*.^36^ observed a continuous increase of isoprene emissions over Asia during 1979–2012. They found a strong correlation (r > 0.90) between isoprene emissions and above-canopy solar radiation, suggesting that enhanced solar radiation intensifies isoprene emissions from terrestrial higher plants over Asia (particularly in China). Similarly, Zhang, *et al*.^72^ have recently reported an increase of biogenic isoprene emissions in northern China during 1982–2010 using the biogenic emission model. It has also been documented that 79% of MeGly may come from biogenic isoprene emissions globally, as inferred from modeling studies^73^. Further, pyruvic (Pyr) and ωC_2_ have been suggested as in-cloud oxidation products of isoprene, which are subsequently oxidized to C_2_^18^. Therefore, it may be possible that biogenic isoprene derived volatile organic precursors (e.g., MeGly) over East Asia/China are taken up by aqueous-phase aerosol particles in the atmosphere and transported to the WNP.

In this study, we found a significant increasing trend (p < 0.05) in the concentrations of MeGly (+0.008% yr^−1^), Pyr (+0.025% yr^−1^) (Fig. 2d and f) and C_2_/MeGly ratios (+0.007% yr^−1^), suggesting the formation of C_2_ from the oxidation of MeGly and Pyr via an intermediate compound, i.e., ωC_2_ (MeGly → Pyr → acetic acid → ωC_2_ → C_2_) in the aqueous-phase^18^. These results suggest that enhanced concentrations of the diacids are probably caused by an increase of biogenic isoprene-derived precursors (i.e., MeGly and Pyr)^74^, followed by the subsequent photochemical oxidation during long-range atmospheric transport over the WNP. A concurrent negative trend in Gly/MeGly (−0.008% yr^−1^; p < 0.05) (Fig. 2h) also suggests an increase of biogenic precursors over the WNP. This point is further supported by our previous study of Boreddy and Kawamura^48^, which reported that a significant increase (p < 0.05) in the concentrations of methanesulfonic acid (MSA^−^; a tracer for biogenic sources) occurred during 2001–2012 over the WNP.

The observed trends of diacids may be not only reflected by changes in emissions/air masses, but also influenced by changes in oxidant levels. To better understand the variations in the trends of oxidant levels over the WNP, we downloaded the monthly mean levels of total columnar ozone (DU) and daily tropospheric columnar NO_2_ (cm^−2^) for the periods from 2002 to 2013 and from 2005 to 2013, respectively, from the NASA website (https://giovanni.gsfc.nasa.gov/) (Figure S3). From Figure S3, it is clear that both oxidant levels (O_3_ and NO_2_) showed significant (p < 0.05) increase in those trends (+0.0004% yr^−1^ and +0.008% yr^−1^, respectively) during the study period, indicating that increased oxidation processes over the WNP lead to increases in the formation of diacids during long-range atmospheric transport.

Based on the above results, we conclude that the enhanced concentrations of diacids over the WNP may be caused due to the increased oxidations of biogenic precursor compounds during long-range atmospheric transport. In contrast, anthropogenic precursors (e.g., Gly) have decreased (or constant) during the study period. It should be noted that all these trends are explained for the whole period (2001–2013); however, seasonal trends may give different results over the WNP because the formations of diacids are very sensitive to the sources of an air mass and the meteorological parameters as reported in Tables S4–S7.

As seen from Tables S4–S7, it is noteworthy that concentrations of total diacids in all seasons showed increases in their trends, except for summer, which showed a decreasing trend (−0.002% yr^−1^; p > 0.05) (Table S6). The declined concentrations of diacids and related compounds in summer are probably due to the pristine air masses, which suggest the negligible local anthropogenic emissions as well as long-range continental outflow over the WNP. It is also seen from Tables S4–S7 that the trend in concentrations of C_9_ showed a decrease in all seasons, except for summer. In summer, the trend of C_9_ showed an increase (+0.001% yr^−1^) during 2001–2013, indicating an importance of oxidation of biogenic unsaturated fatty acids over the WNP, particularly in summer.

### Monthly and seasonal variations

**Figure 3:**
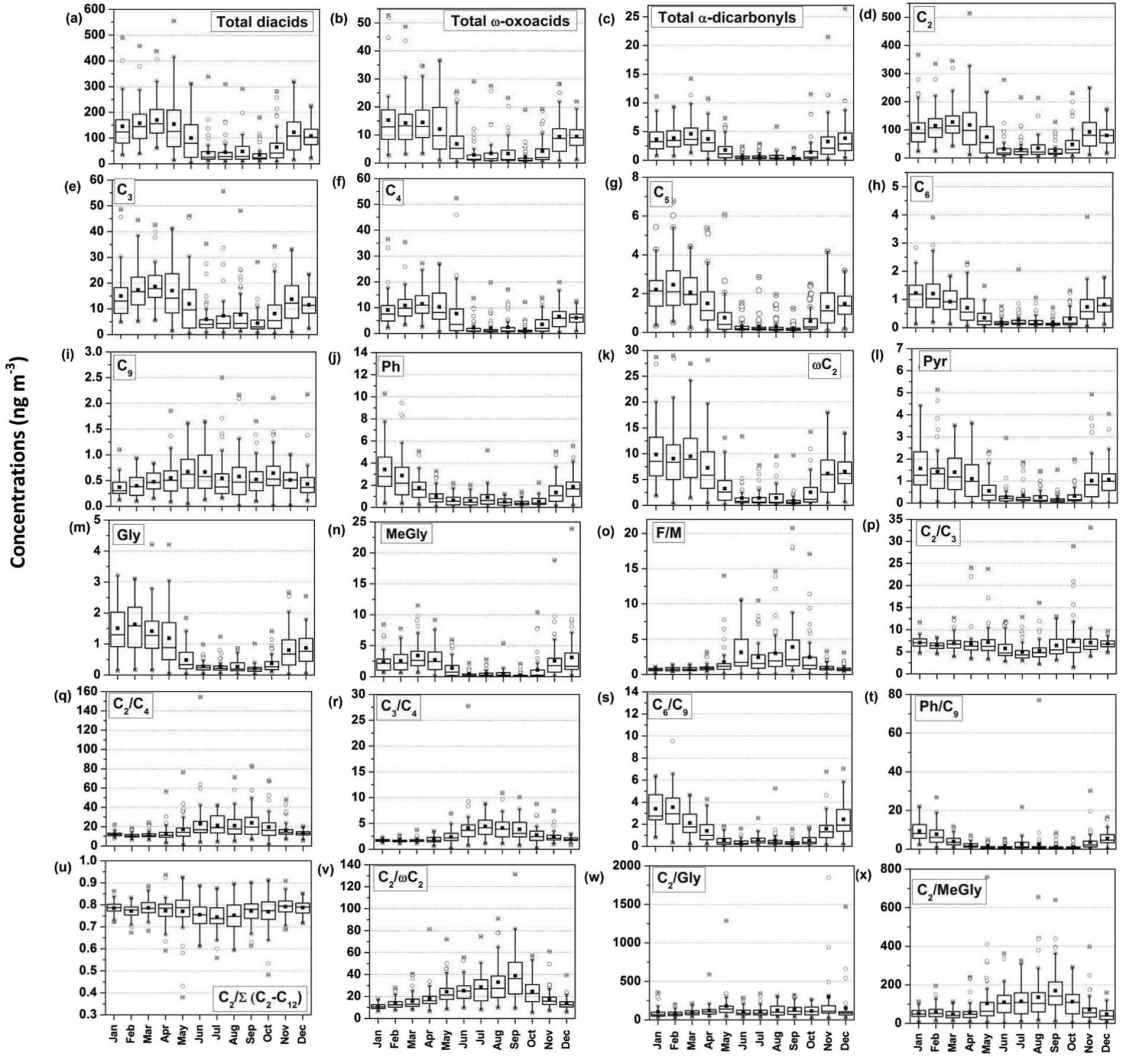
Monthly variations in water-soluble dicarboxylic acids (ng m^−3^) and related compounds (ng m^−3^) and various mass ratios at Chichijima during 2001 to 2013. The horizontal line and dot inside the box indicate maiden and mean, respectively. The vertical hinges represent data points from the lower to the upper quartile (i.e., 25^th^ and 75^th^ percentiles). The whiskers represent data points from the 5^th^ to 95^th^ percentiles. Open circles indicate the outliers.

Figure 3 presents box and whisker plots of monthly variations of diacids, ω-oxocarboxylic acids, pyruvic acid, α-dicarbonyls, and diagnostic mass ratios at Chichijima Island for the period of 2001 to 2013. Almost all the organic compounds showed clear monthly/seasonal variations with higher concentrations in winter/spring under the continental outflow from East Asia and lower concentrations in summer/autumn due to the pristine marine air mass, except for C_9_ (Fig. 3i). As shown in Fig. 3a, concentrations of total diacids are characterized by a gradual increase from autumn to winter, with a peak in early spring (March), and a decrease with a minimum in summer although a small peak was observed in August. Total ω-oxoacids showed a similar seasonal variation (Fig. 3b). The peak in spring is more significant than in summer. On the other hand, total concentrations of α-dicarbonyls gradually increased from late autumn to early spring and then decreased towards the summer months (Fig. 3c). These seasonal variations can be explained primarily by an enhanced Asian outflow in winter/spring with the heterogeneity of air masses and changes in emission strength and meteorology as discussed below.

#### Impact of heterogeneity in air masses

As discussed above, there is a clear seasonal difference in the origin of air masses during the study period at Chichijima Island in the WNP. Such changes in air masses may lead to different effects on the formation of diacids and related compounds as well as the characteristics of chemical properties of aerosols. During winter/spring, the air mass transport is stronger; mostly coming from East Asia with westerly winds. The sampling site is influenced by long-range atmospheric transport of continental air masses, whereas the air masses are originating from the central Pacific via easterly winds during summer/autumn. This heterogeneity in air mass origins may clearly reflect the emission strength of organic aerosols and their precursors with higher concentrations during winter/spring and lower concentrations in summer/autumn over the WNP.

#### Changes in emission strength and formations

Gradual increases of total diacids, ω-oxoacids and α-dicarbonyls during late autumn to early spring are attributable to the combined effect of anthropogenic/biogenic volatile organic compounds (VOCs) emitted from East Asia followed by subsequent oxidation during long-range atmospheric transport. In our previous study, higher concentrations of non-sea salt sulfate (nss-SO_4_^2−^), nitrate (NO_3_^−^), non-sea salt calcium (nss-Ca^2+^) and MSA^−^ were found in winter/spring over the same site under continental influence during the study period^48^. Being consistent with monthly variations of inorganic ion concentrations, C_2_–C_4_ diacids show similar trends with a gradual increase from late autumn to early spring and decrease towards summer months. The seasonal variations of C_6_, Ph, and Gly are characterized by winter maxima and summer minima (Fig. 3h,j and m). Similarly, concentrations of Pyr and MeGly maximized in winter/spring and minimized in summer (Fig. 3l,n). These results demonstrate that, during late autumn to early spring, East Asian emissions of organic acids and their precursors, followed by long-range atmospheric transport, are important factors in controlling the distributions of diacids and related compounds in the WNP. High speed westerly winds also play an important role in causing the highest concentrations of diacids in spring over the WNP (Figure S1b).

On the other hand, lower concentrations of diacids and related compounds in summer suggest a minor contribution either from local emissions over the sampling site or marine emission of diacids and their precursors in the WNP. Mochida, *et al*.^44^ documented that local anthropogenic emissions for diacids are insignificant at Chichijima, based on the lower concentration ratios of benzo*[a]*pyrene (BaP) to (C_2_–C_11_). Therefore, it is reasonable to believe that the observed concentrations of diacids and their precursors during summer may be associated with marine biological sources and are attributable to the oxidation of unsaturated fatty acids^75^. In this connect, the concentrations of C_9_ (aqueous phase photo-oxidation of biogenic unsaturated fatty acids) show a gradual increase from late spring to late autumn with a maximum in June and thereafter show a gradual decrease towards winter and spring months (Fig. 3i). These results suggest that oxidations of biogenic unsaturated fatty acids are important sources of diacids over the WNP in summer/autumn, although their contribution is relatively small compared to those in winter and spring.

#### Influence of meteorological parameters

Meteorological parameters such as wind speed, solar radiation and cloud cover are crucial for understanding the emission strengths of organic compounds and their oxidation processes in the atmosphere. As inferred from Figure S1, solar radiation was maximized in summer and minimized in winter/spring, indicating a strong photochemical oxidation during summer over the WNP. This result was further discussed in terms of seasonal variations in the mass concentration ratios of diacids. Being consistent with solar radiation, F/M, C_2_/C_4_, and C_3_/C_4_ ratios increased gradually from late spring to summer and stayed high until late autumn, and then decreased towards winter (Fig. 3o,q,r). These seasonal changes suggest an enhanced photochemical oxidation, superimposed with changing regional biology and meteorology in summer and important sources of the diacids over the WNP. On the other hand, wind speed and cloud cover were higher during spring and/or winter (Figure S1), suggesting the enhanced processing of precursor compounds associated with Asian outflows during atmospheric long-range transport that leads to higher concentrations of diacids during spring and winter over the WNP. Similar seasonal variations have been found in the mass concentration ratios of C_6_/C_9_ and Ph/C_9_ with higher values in winter and/or spring (Fig. 3s and t). C_2_/ωC_2_ and C_2_/MeGly ratios showed a gradual increase from early spring to autumn with a peak in early autumn, i.e., September (Fig. 3v and x). These ratios, then decreased towards winter. However, ratios of C_2_/Gly did not show any clear seasonal trend during the study period, although ratios are slightly higher in summer and autumn (Fig. 3w). Although precipitation occurs throughout the year over the WNP, it was maximized in summer (Figure S1d); therefore, it is also an important sink for the diacids in addition to photochemical decompositions of oxalate-iron complexes, particularly in summer.

### Source apportionment

**Figure 4:**
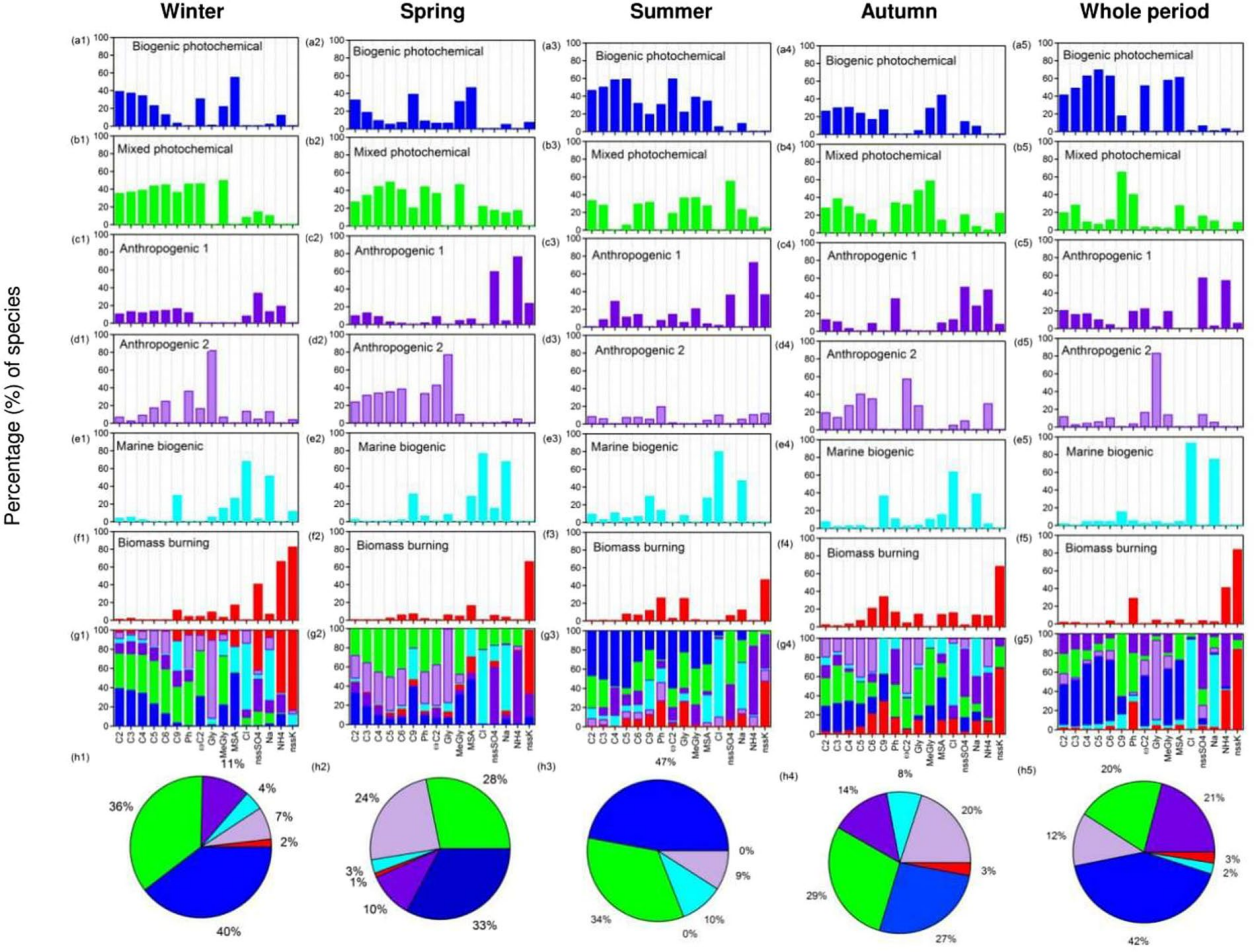
The PMF derived explained variance (%) for the source profiles for the different seasons as well as the whole period (2001–2013) during the study (**a**–**f**). Contributions of each source profile to individual acids (**g**) and oxalic acid (**h**) for the different seasons over the WNP. Each color indicates the different source as mentioned in plots a-f. Therefore, for interpretation of the colors in plots g and h, the reader is referred to the color version of this article.

To quantitatively estimate the contribution of different sources to C_2_ over the WNP, we performed a positive matrix factorization (PMF) analysis for the different seasons and the whole period (2001–2013) as shown in Fig. 4. PMF (version 5.1) is an effective source apportionment receptor model developed by the United States Environmental Protection Agency (U. S. EPA) and is often used in determining the sources of atmospheric aerosols^76, 77^. A complete description of PMF analysis is discussed elsewhere^78–80^. The concentrations of C_2_–C_6_, C_9_, Ph, ωC_2_, Gly, MeGly, and the tracers of water-soluble ions (MSA^−^, Cl^−^, nss-SO_4_^2−^, Na^+^, NH_4_^+^, nss-K^+^) were used as inputs in the PMF analysis. A total of 607 samples were used for this analysis. We identified 6 source profiles such as biogenic photochemical (indicated by blue color), mixed photochemical (green), anthropogenic 1 (purple), anthropogenic 2 (weak purple), marine biogenic (cyan), and biomass burning (red). The derived variations (%) of the species are shown in Fig. 4a–f. The contributions of all sources to the individual diacids for the different seasons as well as for the whole period are shown in Fig. 4g. The detailed descriptions of each PMF resolved-sources are described below for the different seasons over the WNP.

Based on the high variation (%) of loading of MSA^−^, C_2_, C_3_, ωC_2_, MeGly and weak or no loading of C_6_, Ph, and Gly, we identified source 1 as biogenic photochemical 1. Source 2 was identified as mixed photochemical, which was confirmed by a significant loading of all species, which are majorly associated with photochemical oxidation of longer to shorter chain diacids. Source 3 was identified as anthropogenic 1 as evidenced by high loading of nss-SO_4_^2−^ and NH_4_^+^. Source 4 was attributed to anthropogenic 2, because it may be associated with biomass burning-derived VOCs as evidenced by high loading of Gly. Source 5 was considered as marine biogenic unsaturated fatty acids, as evidenced by high loading of Na^+^, Cl^−^, and C_9_. Source 6 was attributed to biomass burning, which was majorly associated with primary emissions, as indicated by high loading of nss-K^+^.

Figure 4h shows the contributions of all sources to C_2_ for the different seasons and the whole period. The biogenic photochemical process is a prominent source of C_2_, whose contribution to C_2_ is highest in summer (47%), followed by winter (40%) and lowest in autumn (27%). The next prominent source is a mixed photochemical process, whose contribution is more abundant during winter (36%), summer (34%) and autumn (29%) and lowest in spring (28%). The contribution of anthropogenic source 1 is higher in autumn (14%) and winter (11%), whereas those of anthropogenic source 2 are higher in spring (24%), autumn (20%) and lower in summer (less than 9%) and winter (7%). Contributions of biomass burning to C_2_ are highest in winter (3%) or autumn (2%) and lowest in summer (<1%), while the contributions of marine biogenic unsaturated fatty acids to C_2_ are highest in summer (10%) followed by autumn (8%) and lowest in spring (3%). Overall, for the whole study period (Fig. 4h5), the contribution of biogenic photochemical process (42%) was the dominant source of C_2_ followed by anthropogenic sources (1 plus 2 contribute ~32%) and mixed photochemical sources (20%). We found that marine biogenic unsaturated fatty acids are important sources for the formation of diacids over the WNP during winter and summer, respectively.

### Conclusions and implications

The 13-year observations of water-soluble diacids and related compounds in marine aerosols in the WNP provided the following findings.

The molecular distributions of diacids showed a predominance of C_2_ followed by C_3_ and C_4_. Seasonal variations of diacids and their precursor compounds showed maxima in winter to spring and minima in summer, except for C_9_, which was maximized in summer. Annual concentrations of total diacids, ω-oxoacids, pyruvic acid and α-dicarbonyls showed continuous increases toward more recent years.

A decrease in anthropogenic emissions is inferred from a decrease in the trends of anthropogenic tracer compounds such as phthalic acid (Ph), adipic acid (C_6_) and glyoxal (Gly), while an increase in biogenic emissions is confirmed from an increase in the concentrations of biogenic tracers including pyruvic acid (Pyr) and methylglyoxal (MeGly) during 2001 to 2013. On the other hand, satellite-derived oxidation levels (total columnar O_3_ and tropospheric columnar NO_2_) showed significant increases during the study period. These results demonstrate that the increased concentrations of diacids over the WNP are probably due to not only the increased biogenic emissions from East Asia but also increased oxidation processes during atmospheric long-range transport, while anthropogenic precursors of the diacids are decreased or constant during the study period over the WNP. These results further support our previous study, which reported the declined and increased concentrations of nss-SO_4_^2−^ and MSA^−^, respectively, over the WNP during 2001–2012. We also found increased concentrations of C_9_ and ωC_9_ in summer, suggesting that marine biogenic unsaturated fatty acids are becoming important sources of diacids over the WNP, particularly, in summer.

These inferences are further supported by PMF analysis, which showed a biogenic photochemical contribution (42%) was a predominant source for C_2_. This is the first study to explain the impact of heterogeneity in air masses on long-term trends of organic acids over the WNP. Therefore, the assessment of future climate effects of East Asian aerosols over the WNP will need continued observations because of the rapid changes in the emission strength of aerosols and their precursors over East Asia. The results of this study should be important for climate modelers, who are interested in radiative forcing calculations over the WNP.

## Methodology

### Collection of aerosol samples

Aerosol samples were collected on a quartz filter (20 × 25 cm, Pallflex 2500QAT-UP) from 2001 to 2013 using a high volume sampler (HVS) with a flow rate of 1 m^3^ min^−1^ at Chichijima Island (27°04′N, 142°13′E) in the WNP (Fig. 1). Before sampling, filters were pre-combusted at 450 °C for three hours. The HVS was set up 5 m above ground level at the Satellite Tracking Centre of Japanese Aerospace Exploration Agency (JAXA, elevation 254 m a.s.l.) in Chichijima Island^45^. 4–6 day integrated samples were collected during the study period. After the sampling, filters were put in a pre-baked (450 °C for 6 hrs) glass bottle with a Teflon lined screw cap and stored at −20 °C prior to the analysis of diacids. A total of 607 aerosol and 61 field blank samples were used in this study. A field blank sample was collected every ten aerosol samples by placing a clean filter in the cartridge of the HVS for 10 sec without the pump running.

### Determination of diacids and related compounds

The collected filter samples were analyzed for diacids and related compounds using the improved method of Kawamura^81^ and Kawamura and Ikushima^1^. Briefly, a portion of each filter sample was extracted three times with 5 ml of organic-free ultrapure water (resistivity of >18 MΩ cm^−1^, Sartorius arium 611 UV) under ultrasonication. The extracts were filtered through a Pasteur pipette packed with quartz wool to remove the filter debris and insoluble materials and placed in a 50 ml pear-shaped flask. The water extracts were pH-adjusted to 8.5–9.0 using a 0.05 M potassium hydroxide (KOH) solution and then concentrated to almost dryness using a rotary evaporator under vacuum. A 14% borontrifluoride in n-butanol solution was added to the extracts and then heated at 100 °C for 1 hour to derivatize the carboxyl and aldehyde groups. The derived dibutyl esters and dibutoxy acetals were extracted with n-hexane and washed three times with ultrapure water (for the removal of polar compounds, including hydrogen fluoride (HF) and boric acid (H_3_BO_4_) derived from the borontrifluoride) and concentrated using a rotary evaporator in a vacuum and nitrogen (N_2_) blow down system. After being dried, a known amount of n-hexane was added to the ester fraction and derivatives were determined using a gas chromatography with a flame ionization detector (GC/FID; Hewlett-Packard, HP6890). Identification of the GC peaks was confirmed by comparing the GC retention times with those of authentic standards and confirmed by mass spectral examination using a GC/mass spectrometry (GC/MS; Thermoquest, Trace MS).

In order to check the recovery, 5 μL of authentic diacids were spiked on the pre-combusted (450 °C for 6 hrs) quartz filters and analyzed like a real sample using the above-mentioned procedure. The recoveries were 85% for oxalic acid, 90% for malonic acid and more than 90% for succinic, glutaric, and adipic acids. The analytical errors in the duplicate analyses are less than 10%. The concentrations of all diacids and related compounds reported in this study have been corrected using the field blanks. The blank levels were less than 5% for the major species measured in the real samples.

Several studies show that positive and negative artifacts can be significant during filter sampling and organic analysis of aerosols^82, 83^. They generally arise from adsorption of gaseous species to the substrate surface and from particle losses on the walls and volatilization of semi-volatile species, respectively. In order to evaluate the potential artifacts, including adsorption or evaporation of collected particles to the gas phase, we performed simultaneous measurements of the diacids in marine aerosols using a HVS and a denuder/filter/denuder system^43^. It is well established that the artifacts due to adsorption or evaporation are to be minor for the denuder system. We found good agreement between the two different techniques, suggesting that the HVS technique is valid for collection of diacids in marine aerosols^43^. This is true because, in general, evaporation loss of particulate organic species is minor compared to gases adsorbed on the filter surfaces^84^. On the other hand, adsorption of gases is limited because diacids are predominantly observed in particle phase^85^. However, it is noteworthy that organic compounds originating from continental regions are much more aged during atmospheric transport toward Chichijima Island; therefore the artifacts may be minor for the diacids. More details about the potential sampling artifacts are described in elsewhere^44^.

### Statistical analysis

To explore the inter-annual difference in the concentrations of the diacids and related compounds, the statistical regression analysis of variations (ANOVA)^86^ was performed by comparing all the data points during the study period. Differences with p < 0.05 were considered to be statistically significant and indicated by a star (*) in the trend analyses as shown in Table 1. This statistical approach is simple, robust and easy to interpret^87^. For example, the sign of the diacid trend depends on the value of the slope of the regression analysis. In this kind of interpretation, when the slope is greater than zero, the trend is positive (increases) whereas the trend is negative (decreases) when the slope is less than zero. When the slope is equal to zero, there is no trend in the diacid concentrations. Uncertainties in the trends are reported as standard errors in the slope of regression lines and variations in the trends (% yr^−1^) are also reported for each organic compound in Table 1.

## Electronic supplementary material

Supporting information
