# Supplementary material for: Long-term (2001–2013) observations of water-soluble dicarboxylic acids and related compounds over the western North Pacific: trends, seasonality and source apportionment
Source: Sci Rep. 2017 Aug 17;7:8518. doi: 10.1038/s41598-017-08745-w (PMC5561035; doi:10.1038/s41598-017-08745-w)
Supplement: Supplementary file 1 — Supporting information [file 41598_2017_8745_MOESM1_ESM.doc]

Supporting Information for

**Long-term (2001-2013) observations of water-soluble dicarboxylic acids and related compounds over the western North Pacific: trends, seasonality and source apportionment**

S.K.R. Boreddy, Kimitaka Kawamura*, and Eri Tachibana

Institute of Low Temperature Science, Hokkaido University, N19, W8, Kita-Ku, Sapporo-060-0819, Japan.

*Now at Chubu Institute for Advanced Studies, Chubu University, Kasugai 487-8501, Japan

**Contents of this file**

Figures S1-S3

Tables S4-S7

**Figure S1.** Temporal variations of meteorological parameters at Chichijima Island in the western North Pacific between 2001 and 2013.

**Figure S2.** Molecular distributions of water-soluble dicarboxylic acids and related compounds in TSP aerosols collected at Chichijima Island during 2001 to 2013. The zoomed figure in box inside the main plot shows the molecular distributions of diacids >C_4_. The horizontal line and dot inside the box indicate maiden and mean, respectively. The vertical hinges represent data points from the lower to the upper quartile (i.e., 25^th^ and 75^th^ percentiles). The whiskers represent data points from the 5^th^ to 95^th^ percentiles.


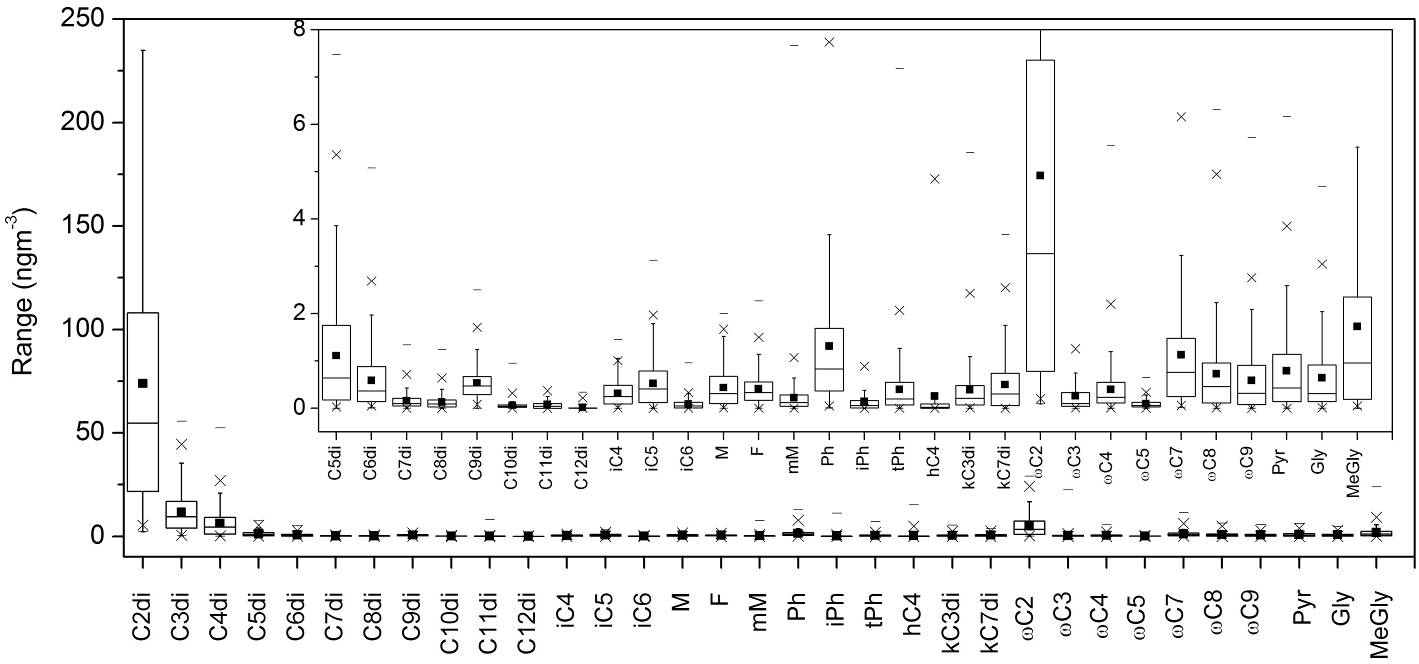


**Figure S3.** Time series area averaged of (a) monthly mean ozone total column (1 deg, DU) during September 2002 to December 2013 (b) daily NO_2_ tropospheric column (0.25 deg; 30% cloud screened; cm^-2^) during 2005 to 2013. Data were downloaded from the NASA website (https://giovanni.gsfc.nasa.gov/) over the region 137.373-145.459° E 18.6914-30.996° N in the WNP.

**Table S4**. Winter time regression statistics (range, mean±SD, and slope) of water-soluble dicarboxylic acids and its related compounds (n=146) in remote marine TSP aerosols collected at Chichijima Island during 2001 to 2013. The symbol, *, indicates the trends are significant at a 95% (p<0.05) confidence level.

| Organic compounds | Range (mean±SD) | Slope (m, diacid year^-1^) | Uncertainty (σm) | Trend (% year^-1^) |
| --- | --- | --- | --- | --- |
| Dicarboxylic acids (ng m^-3^) | | |  |  |
| Normal chain saturated diacids | | |  |  |
| Oxalic, C_2_ | 16.5-366 (110±59) | +0.0023 | 0.0035 | +0.002 |
| Malonic, C_3_ | 2.29-48.6 (14.6±7.84) | -0.00016 | 0.00046 | -0.001 |
| Succinic, C_4_ | 0.84-36.6 (8.67±5.66) | -0.0011 | 0.00033 | -0.001 |
| Glutaric, C_5_ | 0.15-7.48 (2.04±1.27) | -0.00013 | 0.000074 | -0.007 |
| Adipic, C_6_ | 0.11-5.08 (1.08±0.69) | -0.000055 | 0.00004 | -0.005 |
| Pimelic, C_7_ | 0-1.25 (0.22±0.17) | -0.000024* | 0.00001 | -0.011 |
| Suberic, C_8_ | 0-1.24 (0.13±0.14) | -0.000031* | 0.000008 | -0.025 |
| Azelaic, C_9_ | 0.10-2.17 (0.40±0.25) | -0.000058* | 0.000014 | -0.015 |
| Decanedioic, C_10_ | 0-0.28 (0.06±0.05) | -0.00001* | 0.0000026 | -0.017 |
| Undecanedioic, C_11_ | 0-8.03 (0.12±0.66) | +0.000024 | 0.00004 | +0.020 |
| Dodecanedioic, C_12_ | 0-0.25 (0.01±0.03) | -0.0000005 | 0.0000016 | -0.004 |
| Branched chain saturated diacids | | |  |  |
| Methylmalonic, iC_4_ | 0.10-1.45 (0.49±0.21) | -0.000029* | 0.000012 | -0.006 |
| Methylsuccinic, iC_5_ | 0-2.82 (0.87±0.48) | -0.000015 | 0.000028 | -0.002 |
| Methylglutaric, iC_6_ | 0.01-0.96 (0.17±0.12) | -0.000022* | 0.000006 | -0.013 |
| Multi functional saturated diacids | | |  |  |
| Hydroxysuccinic, hC_4_ | 0-5.73 (0.36±1.00) | -0.00014* | 0.000058 | -0.040 |
| Ketomalonic, kC_3_ | 0.06-5.40 (0.70±0.80) | +0.00002 | 0.000048 | +0.003 |
| Ketopimelic, kC_7_ | 0-3.63 (0.76±0.55) | -0.00004 | 0.000032 | -0.005 |
| Unsaturated aliphatic diacids | | |  |  |
| Maleic, M | 0.11-1.82 (0.79±0.37) | -0.000098* | 0.00002 | -0.012 |
| Fumaric, F | 0.13-1.50 (0.55±0.27) | -0.000036* | 0.000016 | -0.007 |
| Methylmaleic, mM | 0-7.66 (0.43±0.78) | -0.0001* | 0.000044 | -0.040 |
| Unsaturated aromatic diacids | | |  |  |
| Phthalic, Ph | 0.17-12.9 (2.74±2.11) | -0.00019 | 0.000125 | -0.007 |
| Isophthalic, iPh | 0-1.92 (0.24±0.27) | +0.000007 | 0.000016 | +0.003 |
| Terephthalic, tPh | 0.01-7.18 (0.79±0.76) | +0.00003 | 0.000045 | +0.004 |
| *Total diacids* | 21.9-490 (136±78.3) | +0.001054 | 0.00467 | +0.001 |
| ω-Oxocarboxylic acids (ng m^-3^) | | |  |  |
| Glyoxylic, ωC_2_ | 0.34-28.9 (8.51±5.25) | -0.000009 | 0.00031 | -0.0001 |
| 3-Oxopropanoic, ωC_3_ | 0.01-22.3 (0.52±1.85) | +0.0001 | 0.00011 | +0.020 |
| 4-Oxobutanoic, ωC_4_ | 0.05-2.54 (0.60±0.44) | +0.00002 | 0.000026 | +0.004 |
| 5-Oxopentanoic, ωC_5_ | 0-0.65 (0.14±0.10) | +0.000013* | 0.000006 | +0.010 |
| 7-Oxoheptanoic, ωC_7_ | 0.11-7.90 (1.37±1.12) | -0.0000198 | 0.000066 | -0.001 |
| 8-Oxooctanoic, ωC_8_ | 0.02-6.16 (0.90±0.80) | -0.0000036 | 0.000047 | -0.0004 |
| 9-Oxononanoic, ωC_9_ | 0-5.72 (1.0±0.81) | +0.000025 | 0.000048 | +0.003 |
| *Total oxoacids* | 1.07-52.7 (13.0±8.83) | +0.00013 | 0.00052 | +0.001 |
| Ketoacid (ng m^-3^) | | |  |  |
| Pyruvic, Pyr | 0-6.16 (1.36-1.03) | +0.0002* | 0.000059 | +0.015 |
| α- Dicarbonyls (ng m^-3^) | | |  |  |
| Glyoxal, Gly | 0.04-4.69 (1.36±0.81) | -0.00021 | 0.0000776 | -0.016 |
| Methylglyoxal, MeGly | 0.14-23.9 (2.70±2.59) | +0.0002 | 0.000153 | +0.009 |
| *Total α-dicarbonyls* | 0.38-26.4 (3.76±2.97) | +0.0004* | 0.00017 | +0.012 |
| Ratios | | |  |  |
| *F/M* | 0.12-3.13 (0.77±0.35) | +0.000075* | 0.000012 | +0.010 |
| *C_2_/C_3_* | 4.54-11.6 (6.83±1.07) | +0.00025* | 0.00006 | +0.004 |
| *C_2_/C_4_* | 5.48-22.2 (12.1±2.96) | +0.00052* | 0.00017 | +0.004 |
| *C_3_/C_4_* | 0.81-2.97 (1.79±0.37) | +0.000012 | 0.00002 | +0.001 |
| *C_2_/∑(C_2_-C_12_)* | 0.67-0.86 (0.78±0.03) | +0.000008* | 0.0000016 | +0.001 |
| *C_2_/ωC_2_* | 5.96-39.4 (12.6±4.54) | +0.00065* | 0.000265 | +0.005 |
| *C_2_/MeGly* | 6.18-194 (52.7±31.9) | +0.0016 | 0.00189 | +0.003 |
| *Gly/MeGly* | 0.04-4.43 (0.77±0.64) | -0.0002* | 0.000056 | -0.025 |

**Table S5**. Spring time regression statistics (range, mean±SD, and slope) of water-soluble dicarboxylic acids and its related compounds (n=154) in remote marine TSP aerosols collected at Chichijima Island during 2001 to 2013. The symbol, *, indicates the trends are significant at a 95% (p<0.05) confidence level.

| **Organic compounds** | **Range (mean±SD)** | Slope (m, diacid year^-1^) | | | | Uncertainty (σm) | | | Trend (% year^-1^) | | | | |
| --- | --- | --- | --- | --- | --- | --- | --- | --- | --- | --- | --- | --- | --- |
| Dicarboxylic acids (ng m^-3^) | | | |  | | | | | | | |  | |
| Normal chain saturated diacids | | |  |  | | |  | | |  | | |  |
| Oxalic, C_2_ | 2.21-514 (106±77.7) | | +0.006 | | 0.004 | | | +0.006 | | |  | | |
| Malonic, C_3_ | 0.34-46.1 (15.9±10.2) | | +0.00043 | | 0.0005 | | | +0.003 | | |  | | |
| Succinic, C_4_ | 0.08±52.4 (9.85±8.01) | | +0.00008 | | 0.00045 | | | +0.008 | | |  | | |
| Glutaric, C_5_ | 0.01-6.07 (1.44±1.20) | | +0.00003 | | 0.00007 | | | +0.002 | | |  | | |
| Adipic, C_6_ | 0.01-2.30 (0.66±0.51) | | -0.00001 | | 0.00003 | | | -0.002 | | |  | | |
| Pimelic, C_7_ | 0-1.34 (0.21±0.20) | | -0.000026* | | 0.000011 | | | -0.012 | | |  | | |
| Suberic, C_8_ | 0-0.96 (0.15±0.15) | | -0.000032* | | 0.000008 | | | -0.022 | | |  | | |
| Azelaic, C_9_ | 0.04-1.85 (0.57±0.32) | | -0.000018 | | 0.000018 | | | -0.003 | | |  | | |
| Decanedioic, C_10_ | 0-0.39 (0.05±0.05) | | -0.000005 | | 0.000003 | | | -0.011 | | |  | | |
| Undecanedioic, C_11_ | 0-0.55 (0.06±0.09) | | -0.000012* | | 0.000005 | | | -0.019 | | |  | | |
| Dodecanedioic, C_12_ | 0-0.11 (0.01±0.02) | | -0.0000003 | | 0.0000011 | | | -0.003 | | |  | | |
| Branched chain saturated diacids | | |  |  | | |  | | | | | | |
| Methylmalonic, iC_4_ | 0-1.06 (0.37±0.25) | | -0.000005 | | 0.000014 | | | -0.001 | | |  | | |
| Methylsuccinic, iC_5_ | 0-3.13 (0.68±0.49) | | +0.000046 | | 0.000027 | | | +0.007 | | |  | | |
| Methylglutaric, iC_6_ | 0-0.61 (0.09±0.08) | | -0.0000001 | | 0.000004 | | | -0.000 | | |  | | |
| Multi functional saturated diacids | | |  |  | | |  | | | | | | |
| Hydroxysuccinic, hC_4_ | 0-15.2 (0.39±1.68) | | -0.00012 | | 0.00009 | | | -0.031 | | |  | | |
| Ketomalonic, kC_3_ | 0-2.58 (0.51±0.47) | | +0.00006* | | 0.000026 | | | +0.012 | | |  | | |
| Ketopimelic, kC_7_ | 0-2.87 (0.72±0.63) | | +0.000012 | | 0.000036 | | | +0.002 | | |  | | |
| Unsaturated aliphatic diacids | | |  |  | | |  | | | | | | |
| Maleic, M | 0-2 (0.54±0.40) | | -0.00006* | | 0.000022 | | | -0.012 | | |  | | |
| Fumaric, F | 0-2.27 (0.47±0.36) | | -0.000037 | | 0.000020 | | | -0.008 | | |  | | |
| Methylmaleic, mM | 0-1.07 (0.23±0.22) | | -0.000053* | | 0.000012 | | | -0.023 | | |  | | |
| Unsaturated aromatic diacids | | |  |  | | |  | | | | | | |
| Phthalic, Ph | 0.03-5.08 (1.14±0.91) | | -0.000041 | | 0.000052 | | | -0.004 | | |  | | |
| Isophthalic, iPh | 0-11.1 (0.21±0.90) | | +0.00002 | | 0.000052 | | | +0.010 | | |  | | |
| Terephthalic, tPh | 0-2.26 (0.42±0.41) | | +0.00002 | | 0.000023 | | | +0.005 | | |  | | |
| *Total diacids* | 2.93-555 (141±96.7) | | +0.0070 | | 0.0055 | | | +0.005 | | |  | | |
| ω-Oxocarboxylic acids (ng m^-3^) | | | |  | | | | | | | |  | |
| Glyoxylic, ωC_2_ | 0.09-28.1 (6.64±5.47) | | -0.0000258 | | 0.0003 | | | -0.0004 | | |  | | |
| 3-Oxopropanoic, ωC_3_ | 0-1.38 (0.32±0.31) | | +0.000084* | | 0.000016 | | | +0.027 | | |  | | |
| 4-Oxobutanoic, ωC_4_ | 0.01-5.56 (0.57±0.65) | | +0.00007* | | 0.000037 | | | +0.013 | | |  | | |
| 5-Oxopentanoic, ωC_5_ | 0-0.35 (0.11±0.08) | | +0.000016* | | 0.0000045 | | | +0.015 | | |  | | |
| 7-Oxoheptanoic, ωC_7_ | 0.05-8.91 (1.73±1.38) | | +0.000056 | | 0.00008 | | | +0.003 | | |  | | |
| 8-Oxooctanoic, ωC_8_ | 0-6.32 (1.07±1.01) | | +0.000055 | | 0.000057 | | | +0.005 | | |  | | |
| 9-Oxononanoic, ωC_9_ | 0-2.89 (0.68±0.62) | | +0.000098* | | 0.000034 | | | +0.015 | | |  | | |
| *Total oxoacids* | 0.21-36.6 (11.1±8.35) | | +0.0003 | | 0.00048 | | | +0.003 | | |  | | |
| Ketoacid (ng m^-3^) | | | |  | | | | | | | |  | |
| Pyruvic, Pyr | 0-3.63 (1.01±0.90) | | +0.0001* | | 0.00005 | | | +0.018 | | |  | | |
| α- Dicarbonyls (ng m^-3^) | | | |  | | | | | | | |  | |
| Glyoxal, Gly | 0.03-4.22 (1.08±0.83) | | -0.000055 | | 0.000075 | | | -0.005 | | |  | | |
| Methylglyoxal, MeGly | 0.01-11.5 (2.50±2.19) | | +0.000062 | | 0.00012 | | | +0.002 | | |  | | |
| *Total α-dicarbonyls* | 0.04-14.2 (3.31±2.70) | | +0.0003 | | 0.00015 | | | +0.009 | | |  | | |
| Ratios | | | |  | | | | | | | |  | |
| *F/M* | 0-14 (1.17±1.14) | | +0.000033 | | 0.00008 | | | +0.003 | | |  | | |
| *C_2_/C_3_* | 3.30-24.0 (6.99±2.98) | | +0.00046* | | 0.000167 | | | +0.007 | | |  | | |
| *C_2_/C_4_* | 0.80-76.4 (13.9±9.11) | | +0.0012* | | 0.00051 | | | +0.009 | | |  | | |
| *C_3_/C_4_* | 0.15-6.92 (2±0.90) | | +0.000014 | | 0.00005 | | | +0.001 | | |  | | |
| *C_2_/∑(C_2_-C_12_)* | 0.38-0.94 (0.78±0.07) | | +0.000006 | | 0.0000038 | | | +0.001 | | |  | | |
| *C_2_/ωC_2_* | 6.72-81.2 (19.5±10.6) | | +0.0017* | | 0.0006 | | | +0.009 | | |  | | |
| *C_2_/MeGly* | 4.55-758 (70.55±83.0) | | +0.011* | | 0.0046 | | | +0.016 | | |  | | |
| *Gly/MeGly* | 0.08-2.47 (0.63±0.43) | | -0.000014* | | 0.000039 | | | -0.002 | | | |  | |

**Table S6**. Summer time regression statistics (range, mean±SD, slope) of water-soluble dicarboxylic acids and its related compounds (n=145) in remote marine TSP aerosols collected at Chichijima Island during 2001 to 2013. The symbol, *, indicates the trends are significant at a 95% (p<0.05) confidence level.

| **Organic compounds** | **Range (mean±SD)** |  | Slope (m, diacid year^-1^) | | Uncertainty (σm) | | Trend (% year^-1^) | | |
| --- | --- | --- | --- | --- | --- | --- | --- | --- | --- |
| Dicarboxylic acids (ng m^-3^) | | | |  | | | |  | |
| Normal chain saturated diacids | |  | |  | |  | | | |
| Oxalic, C_2_ | 3.44-277 (33.4±39.2) |  | -0.0002 | | 0.0023 | | -0.001 | | |
| Malonic, C_3_ | 0.65-55.6 (7.06±8.51) |  | -0.00027 | | 0.0005 | | -0.004 | | |
| Succinic, C_4_ | 0.05-19.8 (2.17±3.23) |  | -0.000018 | | 0.0002 | | -0.001 | | |
| Glutaric, C_5_ | 0-2.85 (0.30±0.40) |  | -0.00001 | | 0.00002 | | -0.003 | | |
| Adipic, C_6_ | 0.05-2.06 (0.22±0.23) |  | -0.000011 | | 0.000014 | | -0.005 | | |
| Pimelic, C_7_ | 0-0.56 (0.09±0.10) |  | -0.000003 | | 0.000006 | | -0.004 | | |
| Suberic, C_8_ | 0-0.47 (0.09±0.10) |  | -0.00003* | | 0.000005 | | -0.038 | | |
| Azelaic, C_9_ | 0.01-2.50 (0.60±0.43) |  | +0.000009 | | 0.000026 | | +0.001 | | |
| Decanedioic, C_10_ | 0-0.91 (0.05±0.09) |  | +0.000004 | | 0.000005 | | +0.007 | | |
| Undecanedioic, C_11_ | 0-1.04 (0.07±0.11) |  | -0.000017* | | 0.000006 | | -0.026 | | |
| Dodecanedioic, C_12_ | 0-0.23 (0.01±0.03) |  | -0.000007* | | 0.000002 | | -0.064 | | |
| Branched chain saturated diacids | |  | |  | |  | | | |
| Methylmalonic, iC_4_ | 0-0.74 (0.12±0.12) |  | -0.000006 | | 0.000007 | | -0.005 | | |
| Methylsuccinic, iC_5_ | 0-1.50 (0.18±0.19) |  | +0.000006 | | 0.000011 | | +0.003 | | |
| Methylglutaric, iC_6_ | 0-0.13 (0.02±0.02) |  | -0.000002 | | 0.0000014 | | -0.009 | | |
| Multi functional saturated diacids | |  | |  | |  | | | |
| Hydroxysuccinic, hC_4_ | 0-3.86 (0.11±0.43) |  | -0.00004 | | 0.000026 | | -0.044 | | |
| Ketomalonic, kC_3_ | 0-1.97 (0.14±0.24) |  | +0.000002 | | 0.000014 | | +0.002 | | |
| Ketopimelic, kC_7_ | 0-3.67 (0.20±0.45) |  | -0.0000038 | | 0.000027 | | -0.002 | | |
| Unsaturated aliphatic diacids | |  | |  | |  | | | |
| Maleic, M | 0-0.77 (0.12±0.12) |  | -0.000025* | | 0.000007 | | -0.020 | | |
| Fumaric, F | 0-1.28 (0.24±0.20) |  | +0.000017 | | 0.000012 | | +0.007 | | |
| Methylmaleic, mM | 0-0.92 (0.09±0.11) |  | -0.000025* | | 0.000006 | | -0.030 | | |
| Unsaturated aromatic diacids | |  | |  | |  | | | |
| Phthalic, Ph | 0.01-5.16 (0.67±0.62) |  | -0.0001* | | 0.00003 | | -0.022 | | |
| Isophthalic, iPh | 0-0.49 (0.05±0.08) |  | +0.000001 | | 0.000005 | | +0.003 | | |
| Terephthalic, tPh | 0-0.94 (0.12±0.14) |  | +0.0000018 | | 0.000008 | | +0.002 | | |
| *Total diacids* | 7.38-338 (46.1±52.2) |  | -0.00078 | | 0.0031 | | -0.002 | | |
| ω-Oxocarboxylic acids (ng m^-3^) | | | |  | | | | |  |
| Glyoxylic, ωC_2_ | 0.18-13.3 (1.44±1.91) |  | -0.000022 | | 0.00011 | | -0.002 | | |
| 3-Oxopropanoic, ωC_3_ | 0-1.26 (0.10±0.19) |  | +0.000015 | | 0.000011 | | +0.015 | | |
| 4-Oxobutanoic, ωC_4_ | 0-2.38 (0.19±0.31) |  | +0.00004* | | 0.00002 | | +0.022 | | |
| 5-Oxopentanoic, ωC_5_ | 0-0.37 (0.05±0.06) |  | +0.000007* | | 0.000003 | | +0.015 | | |
| 7-Oxoheptanoic, ωC_7_ | 0.02-11.6 (0.77±1.43) |  | -0.000034 | | 0.000086 | | -0.004 | | |
| 8-Oxooctanoic, ωC_8_ | 0.01-5.33 (0.48±0.97) |  | -0.0000008 | | 0.000059 | | -0.0002 | | |
| 9-Oxononanoic, ωC_9_ | 0-3.46 (0.27±0.47) |  | +0.000036 | | 0.000028 | | +0.013 | | |
| *Total oxoacids* | 0.40-29.1 (3.29±4.99) |  | +0.000045 | | 0.0003 | | +0.001 | | |
| Ketoacid (ng m^-3^) | | | |  | | | | |  |
| Pyruvic, Pyr | 0-2.95 (0.29±0.42) |  | +0.00007* | | 0.000024 | | +0.025 | | |
| α- Dicarbonyls (ng m^-3^) | | | |  | | | | |  |
| Glyoxal, Gly | 0-1.24 (0.29±0.20) |  | -0.000034 | | 0.00002 | | -0.012 | | |
| Methylglyoxal, MeGly | 0-5.39 (0.43±0.62) |  | -0.000023 | | 0.000037 | | -0.005 | | |
| *Total α-dicarbonyls* | 0.06-5.86 (0.65±0.71) |  | +0.000034 | | 0.000043 | | +0.005 | | |
| Ratios | | | |  | | | | |  |
| *F/M* | 0-14.6 (2.91±2.90) |  | +0.0007* | | 0.00016 | | +0.027 | | |
| *C_2_/C_3_* | 2.18-16.1 (5.35±2.32) |  | +0.00005 | | 0.00014 | | +0.001 | | |
| *C_2_/C_4_* | 3.81-154 (22.0±16.2) |  | +0.00022 | | 0.00098 | | +0.001 | | |
| *C_3_/C_4_* | 0.74-27.7 (4.27±2.73) |  | -0.00007 | | 0.00016 | | -0.002 | | |
| *C_2_/∑(C_2_-C_12_)* | 0.56-0.90 (0.75±0.06) |  | +0.000003* | | 0.0000038 | | +0.0005 | | |
| *C_2_/ωC_2_* | 7.99-91 (29.3±15.03) |  | +0.0021* | | 0.0009 | | +0.007 | | |
| *C_2_/MeGly* | 0-655 (120±93.9) |  | +0.01 | | 0.0056 | | +0.008 | | |
| *Gly/MeGly* | 0-6.71 (1.36±1.01) |  | -0.00007* | | 0.00009 | | -0.005 | | |

**Table S7**. Autumn time regression statistics (range, mean±SD, slope) of water-soluble dicarboxylic acids and its related compounds (n=155) in remote marine TSP aerosols collected at Chichijima Island during 2001 to 2013. The symbol, *, indicates the trends are significant at a 95% (p<0.05) confidence level

| **Organic compounds** | **Range (mean±SD)** |  | Slope (m, diacid year^-1^) | | | Uncertainty (σm) | | Trend (% year^-1^) | | | |
| --- | --- | --- | --- | --- | --- | --- | --- | --- | --- | --- | --- |
| Dicarboxylic acids (ng m^-3^) | | | |  | | | | | | | |
| Normal chain saturated diacids | |  | |  | | |  | | |  | |
| Oxalic, C_2_ | 5.50-249 (56.0±56.1) |  | +0.00392 | | 0.0033 | | | | +0.007 | |  |
| Malonic, C_3_ | 0.28-34.3 (8.79±7.97) |  | +0.00011 | | 0.00048 | | | | +0.001 | |  |
| Succinic, C_4_ | 0.21-19.9 (3.87±4.11) |  | -0.00007 | | 0.00024 | | | | -0.002 | |  |
| Glutaric, C_5_ | 0.02-4.17 (0.70±0.84) |  | -0.000046 | | 0.00005 | | | | -0.007 | |  |
| Adipic, C_6_ | 0.01-3.93 (0.40±0.48) |  | -0.00004 | | 0.00003 | | | | -0.010 | |  |
| Pimelic, C_7_ | 0-0.58 (0.11±0.10) |  | -0.000003 | | 0.000006 | | | | -0.003 | |  |
| Suberic, C_8_ | 0-0.94 (0.14±0.14) |  | -0.000016* | | 0.000008 | | | | -0.012 | |  |
| Azelaic, C_9_ | 0.01-2.10 (0.56±0.32) |  | -0.000024 | | 0.000019 | | | | -0.004 | |  |
| Decanedioic, C_10_ | 0-0.95 (0.08±0.11) |  | -0.0000009 | | 0.000006 | | | | -0.001 | |  |
| Undecanedioic, C_11_ | 0-0.32 (0.07±0.07) |  | -0.000005 | | 0.000004 | | | | -0.008 | |  |
| Dodecanedioic, C_12_ | 0-0.34 (0.02±0.05) |  | -0.000008* | | 0.000003 | | | | -0.042 | |  |
| Branched chain saturated diacids | |  | |  | | |  | | | | |
| Methylmalonic, iC_4_ | 0.04-0.99 (0.27±0.24) |  | -0.000009 | | 0.000014 | | | | -0.003 | |  |
| Methylsuccinic, iC_5_ | 0-2.45 (0.38±0.39) |  | -0.0000057 | | 0.000023 | | | | -0.002 | |  |
| Methylglutaric, iC_6_ | 0-0.36 (0.06±0.07) |  | -0.000006 | | 0.000004 | | | | -0.010 | |  |
| Multi functional saturated diacids | |  | |  | | |  | | | | |
| Hydroxysuccinic, hC_4_ | 0-11.5 (0.18±0.97) |  | -0.00014* | | 0.000057 | | | | -0.080 | |  |
| Ketomalonic, kC_3_ | 0-1.39 (0.21±0.23) |  | +0.0000002 | | 0.000013 | | | | +0.0001 | |  |
| Ketopimelic, kC_7_ | 0-1.97 (0.32±0.41) |  | -0.0000067 | | 0.000025 | | | | -0.002 | |  |
| Unsaturated aliphatic diacids | |  | |  | | |  | | | | |
| Maleic, M | 0.02-1.34 (0.30±0.30) |  | -0.00004* | | 0.000017 | | | | -0.013 | |  |
| Fumaric, F | 0-1.81 (0.39±0.33) |  | -0.00002 | | 0.000019 | | | | -0.005 | |  |
| Methylmaleic, mM | 0-1.33 (0.14±0.20) |  | -0.000039* | | 0.000011 | | | | -0.028 | |  |
| Unsaturated aromatic diacids | |  | |  | | |  | | | | |
| Phthalic, Ph | 0.04-5.07 (0.77±0.82) |  | -0.00005 | | 0.000049 | | | | -0.007 | |  |
| Isophthalic, iPh | 0-0.89 (0.08±0.14) |  | +0.000007 | | 0.000008 | | | | +0.010 | |  |
| Terephthalic, tPh | 0-1.98 (0.26±0.35) |  | +0.0000079 | | 0.000021 | | | | +0.003 | |  |
| *Total diacids* | 7.79-318 (74.1±71.6) |  | +0.0035 | | 0.0043 | | | | +0.005 | |  |
| ω-Oxocarboxylic acids (ng m^-3^) | | | | | | |  | | | |  |
| Glyoxylic, ωC_2_ | 0.12-18 (3.20±3.79) |  | +0.00017 | | 0.00022 | | | | +0.005 | |  |
| 3-Oxopropanoic, ωC_3_ | 0-1.03 (0.13±0.17) |  | +0.000038* | | 0.00001 | | | | +0.031 | |  |
| 4-Oxobutanoic, ωC_4_ | 0.01-1.19 (0.23±0.21) |  | +0.000014 | | 0.000012 | | | | +0.006 | |  |
| 5-Oxopentanoic, ωC_5_ | 0-0.33 (0.07±0.06) |  | +0.000009* | | 0.0000035 | | | | +0.015 | |  |
| 7-Oxoheptanoic, ωC_7_ | 0.06-4.14 (0.67±0.77) |  | +0.00003 | | 0.000046 | | | | +0.005 | |  |
| 8-Oxooctanoic, ωC_8_ | 0-3.60 (0.47±0.60) |  | +0.000025 | | 0.000036 | | | | +0.006 | |  |
| 9-Oxononanoic, ωC_9_ | 0-2.76 (0.43±0.58) |  | +0.000077* | | 0.000034 | | | | +0.018 | |  |
| *Total oxoacids* | 0.35-28.2 (5.19±5.86) |  | +0.00037 | | 0.00035 | | | | +0.007 | |  |
| Ketoacid (ng m^-3^) | | | |  | | |  | | | |  |
| Pyruvic, Pyr | 0-4.93 (0.51±0.74) |  | +0.000154* | | 0.00004 | | | | +0.031 | |  |
| α- Dicarbonyls (ng m^-3^) | | | |  | | |  | | | |  |
| Glyoxal, Gly | 0.02-2.66 (0.48±0.49) |  | +0.0001* | | 0.00004 | | | | +0.022 | |  |
| Methylglyoxal, MeGly | 0.04-18.8 (1.30±2.38) |  | +0.00026 | | 0.000143 | | | | +0.021 | |  |
| *Total α-dicarbonyls* | 0.09-21.5 (1.67±2.71) |  | +0.000426* | | 0.00016 | | | | +0.026 | |  |
| Ratios | | | |  | | |  | | | |  |
| *F/M* | 0-20.7 (2.49±3.32) |  | +0.0003 | | 0.00019 | | | | +0.013 | |  |
| *C_2_/C_3_* | 1.46-33.1 (7.03±3.87) |  | +0.00058* | | 0.00023 | | | | +0.008 | |  |
| *C_2_/C_4_* | 1.56-83.0 (20.0±13.9) |  | +0.0025* | | 0.00081 | | | | +0.013 | |  |
| *C_3_/C_4_* | 0.19-10.1 (3.0±1.72) |  | +0.000198* | | 0.00010 | | | | +0.007 | |  |
| *C_2_/∑(C_2_-C_12_)* | 0.48-0.91 (0.78±0.06) |  | +0.00014* | | 0.0000036 | | | | +0.002 | |  |
| *C_2_/ωC_2_* | 5.48-131 (27.2±18.2) |  | +0.0012 | | 0.00109 | | | | +0.005 | |  |
| *C_2_/MeGly* | 2.55-640 (118±98.3) |  | +0.0053 | | 0.00593 | | | | +0.004 | |  |
| *Gly/MeGly* | 0.01-5.79 (1.17±0.87) |  | -0.00006* | | 0.000082 | | | | -0.005 | |  |
